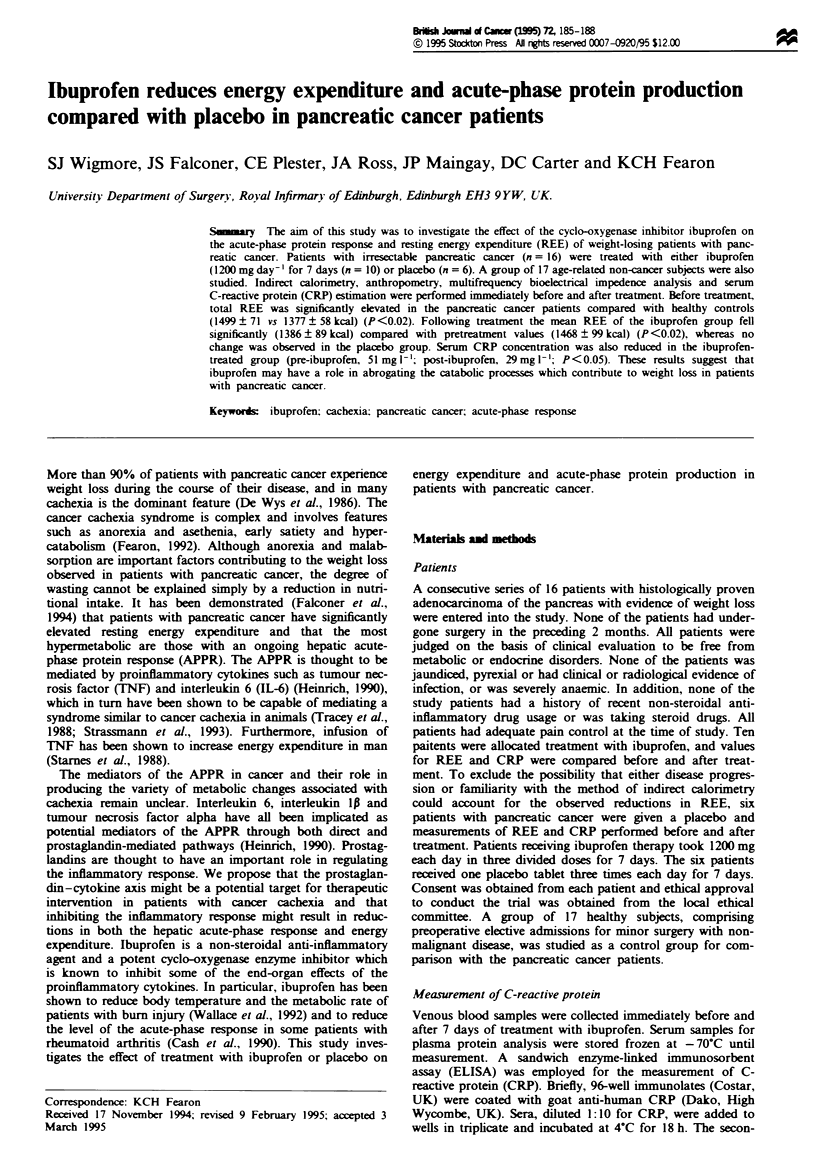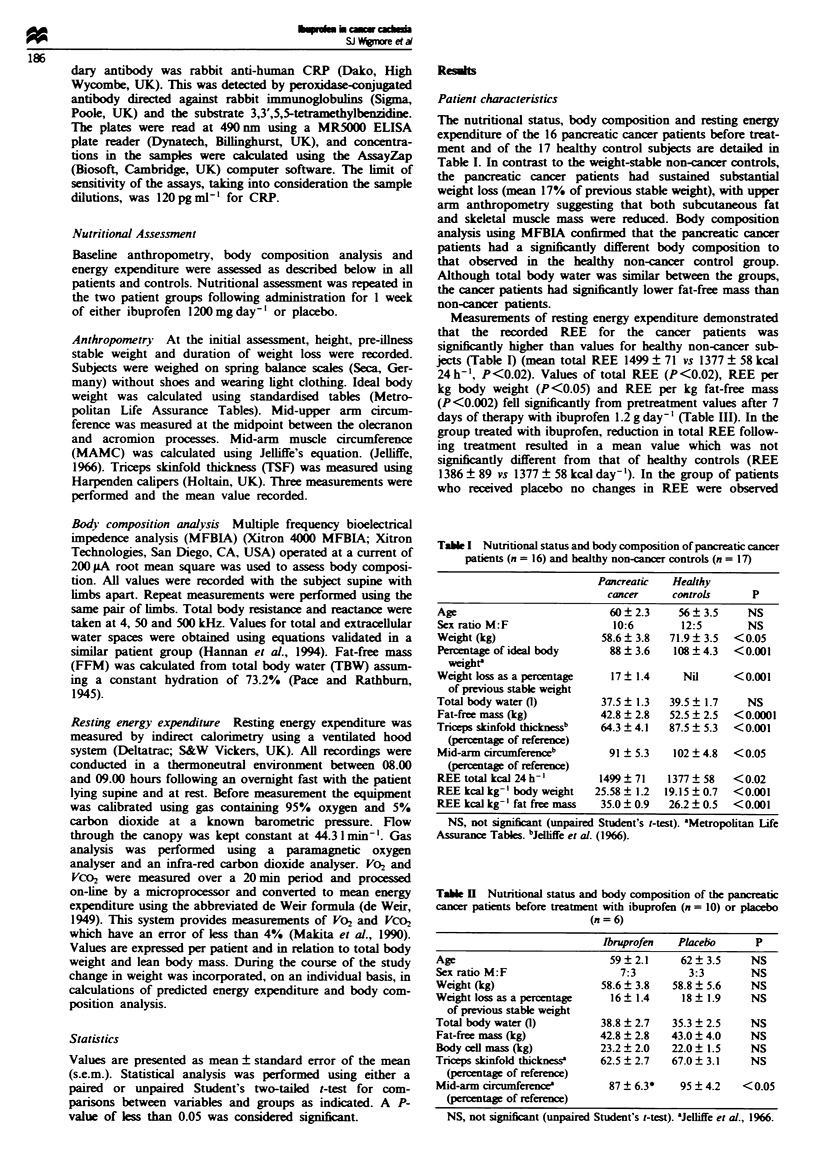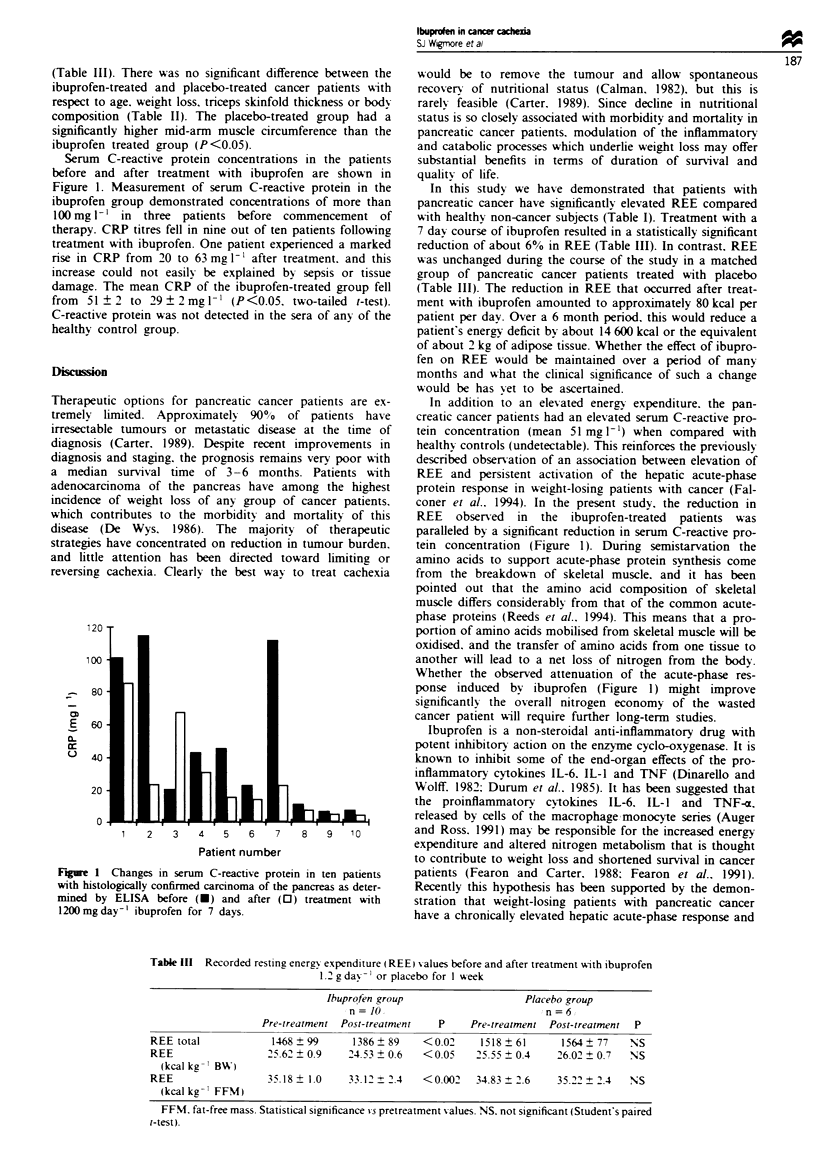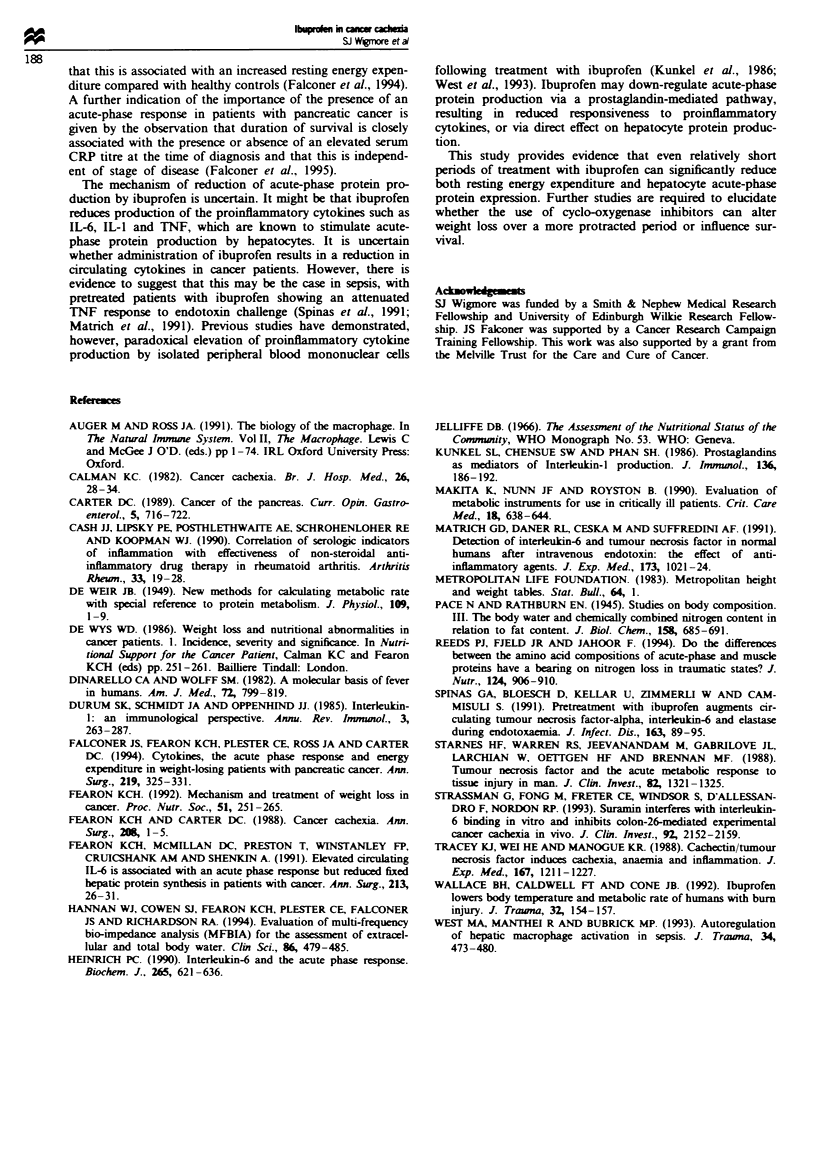# Ibuprofen reduces energy expenditure and acute-phase protein production compared with placebo in pancreatic cancer patients.

**DOI:** 10.1038/bjc.1995.300

**Published:** 1995-07

**Authors:** S. J. Wigmore, J. S. Falconer, C. E. Plester, J. A. Ross, J. P. Maingay, D. C. Carter, K. C. Fearon

**Affiliations:** University Department of Surgery, Royal Infirmary of Edinburgh, UK.

## Abstract

The aim of this study was to investigate the effect of the cyclo-oxygenase inhibitor ibuprofen on the acute-phase protein response and resting energy expenditure (REE) of weight-losing patients with pancreatic cancer. Patients with irresectable pancreatic cancer (n = 16) were treated with either ibuprofen (1200 mg day-1 for 7 days (n = 10) or placebo (n = 6). A group of 17 age-related non-cancer subjects were also studied. Indirect calorimetry, anthropometry, multifrequency bioelectrical impedence analysis and serum C-reactive protein (CRP) estimation were performed immediately before and after treatment. Before treatment, total REE was significantly elevated in the pancreatic cancer patients compared with healthy controls (1499 +/- 71 vs 1377 +/- 58 kcal) (P < 0.02). Following treatment the mean REE of the ibuprofen group fell significantly (1386 +/- 89 kcal) compared with pretreatment values (1468 +/- 99 kcal) (P < 0.02), whereas no change was observed in the placebo group. Serum CRP concentration was also reduced in the ibuprofen-treated group (pre-ibuprofen, 51 mg l-1; post-ibuprofen, 29 mg l-1; P < 0.05). These results suggest that ibuprofen may have a role in abrogating the catabolic processes which contribute to weight loss in patients with pancreatic cancer.


British Journal of Cancer (1995) 72, 185-188

© 1995 Stockton Press All rights reserved 0007-0920/95 $12.00

### Ibuprofen reduces energy expenditure and acute-phase protein production compared with placebo in pancreatic cancer patients

SJ Wigmore, JS Falconer, CE Plester, JA Ross, JP Maingay, DC Carter and KCH Fearon

*University Department of Surgery, Royal Infirmary of Edinburgh, Edinburgh EH3 9YW, UK.*

**Summary** The aim of this study was to investigate the effect of the cyclo-oxygenase inhibitor ibuprofen on the acute-phase protein response and resting energy expenditure (REE) of weight-losing patients with pancreatic cancer. Patients with irresectable pancreatic cancer ($n = 16$) were treated with either ibuprofen (1200 mg day$^{-1}$ for 7 days ($n = 10$) or placebo ($n = 6$). A group of 17 age-related non-cancer subjects were also studied. Indirect calorimetry, anthropometry, multifrequency bioelectrical impedence analysis and serum C-reactive protein (CRP) estimation were performed immediately before and after treatment. Before treatment, total REE was significantly elevated in the pancreatic cancer patients compared with healthy controls (1499 ± 71 *vs* 1377 ± 58 kcal) ($P<0.02$). Following treatment the mean REE of the ibuprofen group fell significantly (1386 ± 89 kcal) compared with pretreatment values (1468 ± 99 kcal) ($P<0.02$), whereas no change was observed in the placebo group. Serum CRP concentration was also reduced in the ibuprofen-treated group (pre-ibuprofen, 51 mg l$^{-1}$; post-ibuprofen, 29 mg l$^{-1}$; $P<0.05$). These results suggest that ibuprofen may have a role in abrogating the catabolic processes which contribute to weight loss in patients with pancreatic cancer.

**Keywords:** ibuprofen; cachexia; pancreatic cancer; acute-phase response

More than 90% of patients with pancreatic cancer experience weight loss during the course of their disease, and in many cachexia is the dominant feature (De Wys *et al.*, 1986). The cancer cachexia syndrome is complex and involves features such as anorexia and asethenia, early satiety and hypercatabolism (Fearon, 1992). Although anorexia and malabsorption are important factors contributing to the weight loss observed in patients with pancreatic cancer, the degree of wasting cannot be explained simply by a reduction in nutritional intake. It has been demonstrated (Falconer *et al.*, 1994) that patients with pancreatic cancer have significantly elevated resting energy expenditure and that the most hypermetabolic are those with an ongoing hepatic acute-phase protein response (APPR). The APPR is thought to be mediated by proinflammatory cytokines such as tumour necrosis factor (TNF) and interleukin 6 (IL-6) (Heinrich, 1990), which in turn have been shown to be capable of mediating a syndrome similar to cancer cachexia in animals (Tracey *et al.*, 1988; Strassmann *et al.*, 1993). Furthermore, infusion of TNF has been shown to increase energy expenditure in man (Starnes *et al.*, 1988).

The mediators of the APPR in cancer and their role in producing the variety of metabolic changes associated with cachexia remain unclear. Interleukin 6, interleukin 1$\beta$ and tumour necrosis factor alpha have all been implicated as potential mediators of the APPR through both direct and prostaglandin-mediated pathways (Heinrich, 1990). Prostaglandins are thought to have an important role in regulating the inflammatory response. We propose that the prostaglandin-cytokine axis might be a potential target for therapeutic intervention in patients with cancer cachexia and that inhibiting the inflammatory response might result in reductions in both the hepatic acute-phase response and energy expenditure. Ibuprofen is a non-steroidal anti-inflammatory agent and a potent cyclo-oxygenase enzyme inhibitor which is known to inhibit some of the end-organ effects of the proinflammatory cytokines. In particular, ibuprofen has been shown to reduce body temperature and the metabolic rate of patients with burn injury (Wallace *et al.*, 1992) and to reduce the level of the acute-phase response in some patients with rheumatoid arthritis (Cash *et al.*, 1990). This study investigates the effect of treatment with ibuprofen or placebo on energy expenditure and acute-phase protein production in patients with pancreatic cancer.

### Materials and methods

### *Patients*

A consecutive series of 16 patients with histologically proven adenocarcinoma of the pancreas with evidence of weight loss were entered into the study. None of the patients had undergone surgery in the preceding 2 months. All patients were judged on the basis of clinical evaluation to be free from metabolic or endocrine disorders. None of the patients was jaundiced, pyrexial or had clinical or radiological evidence of infection, or was severely anaemic. In addition, none of the study patients had a history of recent non-steroidal anti-inflammatory drug usage or was taking steroid drugs. All patients had adequate pain control at the time of study. Ten paitents were allocated treatment with ibuprofen, and values for REE and CRP were compared before and after treatment. To exclude the possibility that either disease progression or familiarity with the method of indirect calorimetry could account for the observed reductions in REE, six patients with pancreatic cancer were given a placebo and measurements of REE and CRP performed before and after treatment. Patients receiving ibuprofen therapy took 1200 mg each day in three divided doses for 7 days. The six patients received one placebo tablet three times each day for 7 days. Consent was obtained from each patient and ethical approval to conduct the trial was obtained from the local ethical committee. A group of 17 healthy subjects, comprising preoperative elective admissions for minor surgery with non-malignant disease, was studied as a control group for comparison with the pancreatic cancer patients.

### *Measurement of C-reactive protein*

Venous blood samples were collected immediately before and after 7 days of treatment with ibuprofen. Serum samples for plasma protein analysis were stored frozen at −70°C until measurement. A sandwich enzyme-linked immunosorbent assay (ELISA) was employed for the measurement of C-reactive protein (CRP). Briefly, 96-well immunolates (Costar, UK) were coated with goat anti-human CRP (Dako, High Wycombe, UK). Sera, diluted 1:10 for CRP, were added to wells in triplicate and incubated at 4°C for 18 h. The secon-

Correspondence: KCH Fearon
Received 17 November 1994; revised 9 February 1995; accepted 3 March 1995

dary antibody was rabbit anti-human CRP (Dako, High Wycombe, UK). This was detected by peroxidase-conjugated antibody directed against rabbit immunoglobulins (Sigma, Poole, UK) and the substrate 3,3',5,5-tetramethylbenzidine. The plates were read at 490 nm using a MR5000 ELISA plate reader (Dynatech, Billinghurst, UK), and concentrations in the samples were calculated using the AssayZap (Biosoft, Cambridge, UK) computer software. The limit of sensitivity of the assays, taking into consideration the sample dilutions, was 120 pg $ml^{-1}$ for CRP.

### *Nutritional Assessment*

Baseline anthropometry, body composition analysis and energy expenditure were assessed as described below in all patients and controls. Nutritional assessment was repeated in the two patient groups following administration for 1 week of either ibuprofen 1200 mg $day^{-1}$ or placebo.

*Anthropometry* At the initial assessment, height, pre-illness stable weight and duration of weight loss were recorded. Subjects were weighed on spring balance scales (Seca, Germany) without shoes and wearing light clothing. Ideal body weight was calculated using standardised tables (Metropolitan Life Assurance Tables). Mid-upper arm circumference was measured at the midpoint between the olecranon and acromion processes. Mid-arm muscle circumference (MAMC) was calculated using Jelliffe's equation. (Jelliffe, 1966). Triceps skinfold thickness (TSF) was measured using Harpenden calipers (Holtain, UK). Three measurements were performed and the mean value recorded.

*Body composition analysis* Multiple frequency bioelectrical impedence analysis (MFBIA) (Xitron 4000 MFBIA; Xitron Technologies, San Diego, CA, USA) operated at a current of 200 μA root mean square was used to assess body composition. All values were recorded with the subject supine with limbs apart. Repeat measurements were performed using the same pair of limbs. Total body resistance and reactance were taken at 4, 50 and 500 kHz. Values for total and extracellular water spaces were obtained using equations validated in a similar patient group (Hannan *et al.*, 1994). Fat-free mass (FFM) was calculated from total body water (TBW) assuming a constant hydration of 73.2% (Pace and Rathburn, 1945).

*Resting energy expenditure* Resting energy expenditure was measured by indirect calorimetry using a ventilated hood system (Deltatrac; S&W Vickers, UK). All recordings were conducted in a thermoneutral environment between 08.00 and 09.00 hours following an overnight fast with the patient lying supine and at rest. Before measurement the equipment was calibrated using gas containing 95% oxygen and 5% carbon dioxide at a known barometric pressure. Flow through the canopy was kept constant at 44.3 l $min^{-1}$. Gas analysis was performed using a paramagnetic oxygen analyser and an infra-red carbon dioxide analyser. $Vo_2$ and $Vco_2$ were measured over a 20 min period and processed on-line by a microprocessor and converted to mean energy expenditure using the abbreviated de Weir formula (de Weir, 1949). This system provides measurements of $Vo_2$ and $Vco_2$ which have an error of less than 4% (Makita *et al.*, 1990). Values are expressed per patient and in relation to total body weight and lean body mass. During the course of the study change in weight was incorporated, on an individual basis, in calculations of predicted energy expenditure and body composition analysis.

### *Statistics*

Values are presented as mean ± standard error of the mean (s.e.m.). Statistical analysis was performed using either a paired or unpaired Student's two-tailed *t*-test for comparisons between variables and groups as indicated. A *P*-value of less than 0.05 was considered significant.

### Results

### *Patient characteristics*

The nutritional status, body composition and resting energy expenditure of the 16 pancreatic cancer patients before treatment and of the 17 healthy control subjects are detailed in Table I. In contrast to the weight-stable non-cancer controls, the pancreatic cancer patients had sustained substantial weight loss (mean 17% of previous stable weight), with upper arm anthropometry suggesting that both subcutaneous fat and skeletal muscle mass were reduced. Body composition analysis using MFBIA confirmed that the pancreatic cancer patients had a significantly different body composition to that observed in the healthy non-cancer control group. Although total body water was similar between the groups, the cancer patients had significantly lower fat-free mass than non-cancer patients.

Measurements of resting energy expenditure demonstrated that the recorded REE for the cancer patients was significantly higher than values for healthy non-cancer subjects (Table I) (mean total REE 1499 ± 71 *vs* 1377 ± 58 kcal 24 $h^{-1}$, $P<0.02$). Values of total REE ($P<0.02$), REE per kg body weight ($P<0.05$) and REE per kg fat-free mass ($P<0.002$) fell significantly from pretreatment values after 7 days of therapy with ibuprofen 1.2 g $day^{-1}$ (Table III). In the group treated with ibuprofen, reduction in total REE following treatment resulted in a mean value which was not significantly different from that of healthy controls (REE 1386 ± 89 *vs* 1377 ± 58 kcal $day^{-1}$). In the group of patients who received placebo no changes in REE were observed

**Table I** Nutritional status and body composition of pancreatic cancer patients ($n = 16$) and healthy non-cancer controls ($n = 17$)

| | *Pancreatic cancer* | *Healthy controls* | P |
|---|---|---|---|
| Age | 60 ± 2.3 | 56 ± 3.5 | NS |
| Sex ratio M:F | 10:6 | 12:5 | NS |
| Weight (kg) | 58.6 ± 3.8 | 71.9 ± 3.5 | <0.05 |
| Percentage of ideal body weight[a] | 88 ± 3.6 | 108 ± 4.3 | <0.001 |
| Weight loss as a percentage of previous stable weight | 17 ± 1.4 | Nil | <0.001 |
| Total body water (l) | 37.5 ± 1.3 | 39.5 ± 1.7 | NS |
| Fat-free mass (kg) | 42.8 ± 2.8 | 52.5 ± 2.5 | <0.0001 |
| Triceps skinfold thickness[b] (percentage of reference) | 64.3 ± 4.1 | 87.5 ± 5.3 | <0.001 |
| Mid-arm circumference[b] (percentage of reference) | 91 ± 5.3 | 102 ± 4.8 | <0.05 |
| REE total kcal 24 $h^{-1}$ | 1499 ± 71 | 1377 ± 58 | <0.02 |
| REE kcal $kg^{-1}$ body weight | 25.58 ± 1.2 | 19.15 ± 0.7 | <0.001 |
| REE kcal $kg^{-1}$ fat free mass | 35.0 ± 0.9 | 26.2 ± 0.5 | <0.001 |

NS, not significant (unpaired Student's *t*-test). [a]Metropolitan Life Assurance Tables. [b]Jelliffe *et al.* (1966).

**Table II** Nutritional status and body composition of the pancreatic cancer patients before treatment with ibuprofen ($n = 10$) or placebo ($n = 6$)

| | *Ibruprofen* | *Placebo* | P |
|---|---|---|---|
| Age | 59 ± 2.1 | 62 ± 3.5 | NS |
| Sex ratio M:F | 7:3 | 3:3 | NS |
| Weight (kg) | 58.6 ± 3.8 | 58.8 ± 5.6 | NS |
| Weight loss as a percentage of previous stable weight | 16 ± 1.4 | 18 ± 1.9 | NS |
| Total body water (l) | 38.8 ± 2.7 | 35.3 ± 2.5 | NS |
| Fat-free mass (kg) | 42.8 ± 2.8 | 43.0 ± 4.0 | NS |
| Body cell mass (kg) | 23.2 ± 2.0 | 22.0 ± 1.5 | NS |
| Triceps skinfold thickness[a] (percentage of reference) | 62.5 ± 2.7 | 67.0 ± 3.1 | NS |
| Mid-arm circumference[a] (percentage of reference) | 87 ± 6.3* | 95 ± 4.2 | <0.05 |

NS, not significant (unpaired Student's *t*-test). [a]Jelliffe *et al.*, 1966.

(Table III). There was no significant difference between the ibuprofen-treated and placebo-treated cancer patients with respect to age, weight loss, triceps skinfold thickness or body composition (Table II). The placebo-treated group had a significantly higher mid-arm muscle circumference than the ibuprofen treated group ($P<0.05$).

Serum C-reactive protein concentrations in the patients before and after treatment with ibuprofen are shown in Figure 1. Measurement of serum C-reactive protein in the ibuprofen group demonstrated concentrations of more than 100 mg $l^{-1}$ in three patients before commencement of therapy. CRP titres fell in nine out of ten patients following treatment with ibuprofen. One patient experienced a marked rise in CRP from 20 to 63 mg $l^{-1}$ after treatment, and this increase could not easily be explained by sepsis or tissue damage. The mean CRP of the ibuprofen-treated group fell from $51 \pm 2$ to $29 \pm 2$ mg $l^{-1}$ ($P<0.05$, two-tailed *t*-test). C-reactive protein was not detected in the sera of any of the healthy control group.

### Discussion

Therapeutic options for pancreatic cancer patients are extremely limited. Approximately 90% of patients have irresectable tumours or metastatic disease at the time of diagnosis (Carter, 1989). Despite recent improvements in diagnosis and staging, the prognosis remains very poor with a median survival time of 3–6 months. Patients with adenocarcinoma of the pancreas have among the highest incidence of weight loss of any group of cancer patients, which contributes to the morbidity and mortality of this disease (De Wys, 1986). The majority of therapeutic strategies have concentrated on reduction in tumour burden, and little attention has been directed toward limiting or reversing cachexia. Clearly the best way to treat cachexia would be to remove the tumour and allow spontaneous recovery of nutritional status (Calman, 1982), but this is rarely feasible (Carter, 1989). Since decline in nutritional status is so closely associated with morbidity and mortality in pancreatic cancer patients, modulation of the inflammatory and catabolic processes which underlie weight loss may offer substantial benefits in terms of duration of survival and quality of life.

In this study we have demonstrated that patients with pancreatic cancer have significantly elevated REE compared with healthy non-cancer subjects (Table I). Treatment with a 7 day course of ibuprofen resulted in a statistically significant reduction of about 6% in REE (Table III). In contrast, REE was unchanged during the course of the study in a matched group of pancreatic cancer patients treated with placebo (Table III). The reduction in REE that occurred after treatment with ibuprofen amounted to approximately 80 kcal per patient per day. Over a 6 month period, this would reduce a patient's energy deficit by about 14 600 kcal or the equivalent of about 2 kg of adipose tissue. Whether the effect of ibuprofen on REE would be maintained over a period of many months and what the clinical significance of such a change would be has yet to be ascertained.

In addition to an elevated energy expenditure, the pancreatic cancer patients had an elevated serum C-reactive protein concentration (mean 51 mg $l^{-1}$) when compared with healthy controls (undetectable). This reinforces the previously described observation of an association between elevation of REE and persistent activation of the hepatic acute-phase protein response in weight-losing patients with cancer (Falconer *et al.*, 1994). In the present study, the reduction in REE observed in the ibuprofen-treated patients was paralleled by a significant reduction in serum C-reactive protein concentration (Figure 1). During semistarvation the amino acids to support acute-phase protein synthesis come from the breakdown of skeletal muscle, and it has been pointed out that the amino acid composition of skeletal muscle differs considerably from that of the common acute-phase proteins (Reeds *et al.*, 1994). This means that a proportion of amino acids mobilised from skeletal muscle will be oxidised, and the transfer of amino acids from one tissue to another will lead to a net loss of nitrogen from the body. Whether the observed attenuation of the acute-phase response induced by ibuprofen (Figure 1) might improve significantly the overall nitrogen economy of the wasted cancer patient will require further long-term studies.

Ibuprofen is a non-steroidal anti-inflammatory drug with potent inhibitory action on the enzyme cyclo-oxygenase. It is known to inhibit some of the end-organ effects of the pro-inflammatory cytokines IL-6, IL-1 and TNF (Dinarello and Wolff, 1982; Durum *et al.*, 1985). It has been suggested that the proinflammatory cytokines IL-6, IL-1 and TNF-$\alpha$, released by cells of the macrophage/monocyte series (Auger and Ross, 1991) may be responsible for the increased energy expenditure and altered nitrogen metabolism that is thought to contribute to weight loss and shortened survival in cancer patients (Fearon and Carter, 1988; Fearon *et al.*, 1991). Recently this hypothesis has been supported by the demonstration that weight-losing patients with pancreatic cancer have a chronically elevated hepatic acute-phase response and

**Figure 1** Changes in serum C-reactive protein in ten patients with histologically confirmed carcinoma of the pancreas as determined by ELISA before (■) and after (□) treatment with 1200 mg day$^{-1}$ ibuprofen for 7 days.

**Table III** Recorded resting energy expenditure (REE) values before and after treatment with ibuprofen 1.2 g day$^{-1}$ or placebo for 1 week

| | *Ibuprofen group* (n = *10*) | | | *Placebo group* (n = *6*) | | |
|---|---|---|---|---|---|---|
| | *Pre-treatment* | *Post-treatment* | P | *Pre-treatment* | *Post-treatment* | P |
| REE total | 1468 ± 99 | 1386 ± 89 | <0.02 | 1518 ± 61 | 1564 ± 77 | NS |
| REE (kcal kg$^{-1}$ BW) | 25.62 ± 0.9 | 24.53 ± 0.6 | <0.05 | 25.55 ± 0.4 | 26.02 ± 0.7 | NS |
| REE (kcal kg$^{-1}$ FFM) | 35.18 ± 1.0 | 33.12 ± 2.4 | <0.002 | 34.83 ± 2.6 | 35.22 ± 2.4 | NS |

FFM, fat-free mass. Statistical significance *vs* pretreatment values. NS, not significant (Student's paired *t*-test).

that this is associated with an increased resting energy expenditure compared with healthy controls (Falconer et al., 1994). A further indication of the importance of the presence of an acute-phase response in patients with pancreatic cancer is given by the observation that duration of survival is closely associated with the presence or absence of an elevated serum CRP titre at the time of diagnosis and that this is independent of stage of disease (Falconer et al., 1995).

The mechanism of reduction of acute-phase protein production by ibuprofen is uncertain. It might be that ibuprofen reduces production of the proinflammatory cytokines such as IL-6, IL-1 and TNF, which are known to stimulate acute-phase protein production by hepatocytes. It is uncertain whether administration of ibuprofen results in a reduction in circulating cytokines in cancer patients. However, there is evidence to suggest that this may be the case in sepsis, with pretreated patients with ibuprofen showing an attenuated TNF response to endotoxin challenge (Spinas et al., 1991; Matrich et al., 1991). Previous studies have demonstrated, however, paradoxical elevation of proinflammatory cytokine production by isolated peripheral blood mononuclear cells following treatment with ibuprofen (Kunkel et al., 1986; West et al., 1993). Ibuprofen may down-regulate acute-phase protein production via a prostaglandin-mediated pathway, resulting in reduced responsiveness to proinflammatory cytokines, or via direct effect on hepatocyte protein production.

This study provides evidence that even relatively short periods of treatment with ibuprofen can significantly reduce both resting energy expenditure and hepatocyte acute-phase protein expression. Further studies are required to elucidate whether the use of cyclo-oxygenase inhibitors can alter weight loss over a more protracted period or influence survival.

**Acknowledgements**

SJ Wigmore was funded by a Smith & Nephew Medical Research Fellowship and University of Edinburgh Wilkie Research Fellowship. JS Falconer was supported by a Cancer Research Campaign Training Fellowship. This work was also supported by a grant from the Melville Trust for the Care and Cure of Cancer.

**References**

AUGER M AND ROSS JA. (1991). The biology of the macrophage. In *The Natural Immune System*. Vol II, *The Macrophage*. Lewis C and McGee J O'D. (eds.) pp 1–74. IRL Oxford University Press: Oxford.

CALMAN KC. (1982). Cancer cachexia. *Br. J. Hosp. Med.*, **26**, 28–34.

CARTER DC. (1989). Cancer of the pancreas. *Curr. Opin. Gastroenterol.*, **5**, 716–722.

CASH JJ, LIPSKY PE, POSTHLETHWAITE AE, SCHROHENLOHER RE AND KOOPMAN WJ. (1990). Correlation of serologic indicators of inflammation with effectiveness of non-steroidal anti-inflammatory drug therapy in rheumatoid arthritis. *Arthritis Rheum.*, **33**, 19–28.

DE WEIR JB. (1949). New methods for calculating metabolic rate with special reference to protein metabolism. *J. Physiol.*, **109**, 1–9.

DE WYS WD. (1986). Weight loss and nutritional abnormalities in cancer patients. 1. Incidence, severity and significance. In *Nutritional Support for the Cancer Patient*, Calman KC and Fearon KCH (eds) pp. 251–261. Bailliere Tindall: London.

DINARELLO CA AND WOLFF SM. (1982). A molecular basis of fever in humans. *Am. J. Med.*, **72**, 799–819.

DURUM SK, SCHMIDT JA AND OPPENHIND JJ. (1985). Interleukin-1: an immunological perspective. *Annu. Rev. Immunol.*, **3**, 263–287.

FALCONER JS, FEARON KCH, PLESTER CE, ROSS JA AND CARTER DC. (1994). Cytokines, the acute phase response and energy expenditure in weight-losing patients with pancreatic cancer. *Ann. Surg.*, **219**, 325–331.

FEARON KCH. (1992). Mechanism and treatment of weight loss in cancer. *Proc. Nutr. Soc.*, **51**, 251–265.

FEARON KCH AND CARTER DC. (1988). Cancer cachexia. *Ann. Surg.*, **208**, 1–5.

FEARON KCH, MCMILLAN DC, PRESTON T, WINSTANLEY FP, CRUICSHANK AM AND SHENKIN A. (1991). Elevated circulating IL-6 is associated with an acute phase response but reduced fixed hepatic protein synthesis in patients with cancer. *Ann. Surg.*, **213**, 26–31.

HANNAN WJ, COWEN SJ, FEARON KCH, PLESTER CE, FALCONER JS AND RICHARDSON RA. (1994). Evaluation of multi-frequency bio-impedance analysis (MFBIA) for the assessment of extracellular and total body water. *Clin Sci.*, **86**, 479–485.

HEINRICH PC. (1990). Interleukin-6 and the acute phase response. *Biochem. J.*, **265**, 621–636.

JELLIFFE DB. (1966). *The Assessment of the Nutritional Status of the Community*, WHO Monograph No. 53. WHO: Geneva.

KUNKEL SL, CHENSUE SW AND PHAN SH. (1986). Prostaglandins as mediators of Interleukin-1 production. *J. Immunol.*, **136**, 186–192.

MAKITA K, NUNN JF AND ROYSTON B. (1990). Evaluation of metabolic instruments for use in critically ill patients. *Crit. Care Med.*, **18**, 638–644.

MATRICH GD, DANER RL, CESKA M AND SUFFREDINI AF. (1991). Detection of interleukin-6 and tumour necrosis factor in normal humans after intravenous endotoxin: the effect of anti-inflammatory agents. *J. Exp. Med.*, **173**, 1021–24.

METROPOLITAN LIFE FOUNDATION. (1983). Metropolitan height and weight tables. *Stat. Bull.*, **64**, 1.

PACE N AND RATHBURN EN. (1945). Studies on body composition. III. The body water and chemically combined nitrogen content in relation to fat content. *J. Biol. Chem.*, **158**, 685–691.

REEDS PJ, FJELD JR AND JAHOOR F. (1994). Do the differences between the amino acid compositions of acute-phase and muscle proteins have a bearing on nitrogen loss in traumatic states? *J. Nutr.*, **124**, 906–910.

SPINAS GA, BLOESCH D, KELLAR U, ZIMMERLI W AND CAMMISULI S. (1991). Pretreatment with ibuprofen augments circulating tumour necrosis factor-alpha, interleukin-6 and elastase during endotoxaemia. *J. Infect. Dis.*, **163**, 89–95.

STARNES HF, WARREN RS, JEEVANANDAM M, GABRILOVE JL, LARCHIAN W, OETTGEN HF AND BRENNAN MF. (1988). Tumour necrosis factor and the acute metabolic response to tissue injury in man. *J. Clin. Invest.*, **82**, 1321–1325.

STRASSMAN G, FONG M, FRETER CE, WINDSOR S, D'ALLESSANDRO F, NORDON RP. (1993). Suramin interferes with interleukin-6 binding in vitro and inhibits colon-26-mediated experimental cancer cachexia in vivo. *J. Clin. Invest.*, **92**, 2152–2159.

TRACEY KJ, WEI HE AND MANOGUE KR. (1988). Cachectin/tumour necrosis factor induces cachexia, anaemia and inflammation. *J. Exp. Med.*, **167**, 1211–1227.

WALLACE BH, CALDWELL FT AND CONE JB. (1992). Ibuprofen lowers body temperature and metabolic rate of humans with burn injury. *J. Trauma*, **32**, 154–157.

WEST MA, MANTHEI R AND BUBRICK MP. (1993). Autoregulation of hepatic macrophage activation in sepsis. *J. Trauma*, **34**, 473–480.